# Volatile compound profiling from soybean oil in the heating process

**DOI:** 10.1002/fsn3.1401

**Published:** 2020-01-14

**Authors:** Lin Xiao, Chongwei Li, Duo Chai, Yan Chen, Zhenyu Wang, Xianbing Xu, Yi Wang, Yufeng Geng, Liang Dong

**Affiliations:** ^1^ School of Food Science and Technology Dalian Polytechnic University Dalian Liaoning; ^2^ National Engineering Research Center of Seafood Dalian Liaoning; ^3^ Engineering Research Center of Agricultural Microbiology Technology Ministry of Education Heilongjiang University Harbin Heilongjiang; ^4^ School of Bioengineering Dalian Polytechnic University Dalian Liaoning

**Keywords:** cooking, heating process, soybean oil, volatile compound

## Abstract

Soybean oil heating or cooking is a very complicated process. In order to better understand the composition of the volatile compounds from soybean oil during heating process, volatile profiling was carried out through vacuum‐assisted headspace solid‐phase microextraction combined with GC‐MS. As a result, a total of 72 volatile compounds were detected and identified during this process, including aldehydes (27), alcohols (14), ketones (10), furans (6), aromatic compounds (9), acids, and esters (6). And the forming temperature of each volatile was determined. Results show most of volatile aldehydes and alcohols were formed at 120°C leading to release off‐flavor largely, which was considered as a critical temperature point for the formation of soybean oil flavor during the whole heating process. Meanwhile, ketones and furans were formed at 150°C, and acids were detected at 180°C. The content of most volatile compounds increased significantly with the temperature raised. Simultaneously, results of principal component analysis demonstrate that flavor characteristics of soybean oil have a big difference between higher and lower temperature in the heating process.

## Graphical abstract

The forming temperature of each volatile from soybean oil was finally determined in this research. It provides a theoretical possibility to modulate flavor production by controlling the temperature in the heating process.

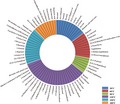

## 1 INTRODUCTION

Deep‐fat frying using edible vegetable oil is a common food cooking method globally, especially in China (Wu & Chen, 1992). Soybean oil is one kind of edible vegetable oils, which is commonly used in heating process and a major source of fried food flavor (Yeboah, Mitei, Ngila, Wessjohann, & Schmidt, 2012). Autoxidation of unsaturated fatty acid in soybean oil is considered as the main source of volatile compounds in heating process, which results in forming desired and undesired flavor. Moreover, the undesired flavor causes the rancidity problems of the fried product (Katragadda, Fullana, Sidhu, & Carbonell‐Barrachina, 2010; Wu & Chen, 1992). Therefore, heating process is a crucial operation process for flavor forming of the frying oil and fried food.

The heating of soybean oil causes a series of chemical reactions which destroy the structure of unsaturated fatty acids and reduce oil quality. Studies have shown that when the oil temperature reaches 250°C, the content of unsaturated fatty acids decreases, while that of transfatty acids gradually increase (Filip, Fink, Hribar, & Vidrih, 2010; Hou, Wang, Wang, Xu, & Zhang, 2012; Moreno, Olivares, López, Adelantado, & Reig, 1999). Excessive intake of transfatty acids can cause a rise in serum total cholesterol and low‐density lipoprotein cholesterol (Mensink & Katan, 1990), which may increase the risk of coronary heart disease (Listed, 1995; Watts, Jackson, Burke, & Lewis, 1996; Xu et al., 2006) and cardiovascular diseases as well as diabetes (Mozaffarian, 2006).

In recent years, researchers paid much attention on studying the composition and characteristics of the off‐flavor in frying oil and fried food products. They found a large number of aldehydes in the gases of cooking oil and fried foods, for example, hexanal, heptanal, and pentanal, which causes unpleasant flavor and reduces the self‐life of the fried products (Fullana, Carbonell‐Barrachina, & Sidhu, 2004; Katragadda et al., 2010; Zhu, Wang, Zhu, & M. K. G., 2001). But to date, the flavor characteristics of soybean oil throughout the whole heating process has not been studied. Therefore, the purpose of this study is to analyze the volatile compositions of soybean oil generated at different temperatures during the heating process, to determine specific temperatures point at which different volatile compounds generated during heating, and to provide a theoretical basis for soybean oil flavor forming.

## 2 MATERIAL AND METHODS

### 2.1 Materials

One kilogram of soybean seeds (HN‐48) was purchased from a local market in Dalian, Liaoning, China. The oil was extracted from the seeds using an expeller machine (HDC, Model LTP205). The extracted oils were filtered and stored in sealed glass bottles in the refrigerator (4°C) for further analysis. Standard chemicals: analytical grade (pentanal, hexanal, heptanal, octanal, nonanal, decanal, 1‐penten‐3‐ol, pentanol, hexanol, heptanol, octanol, toluene, ethyl benzene, p‐xylene, styrene, 2‐pentylfuran, and C4–C20 n‐alkanes) were purchased from Sigma‐Aldrich.

### 2.2 Vacuum‐assisted HS‐SPME

**Figure 1:**
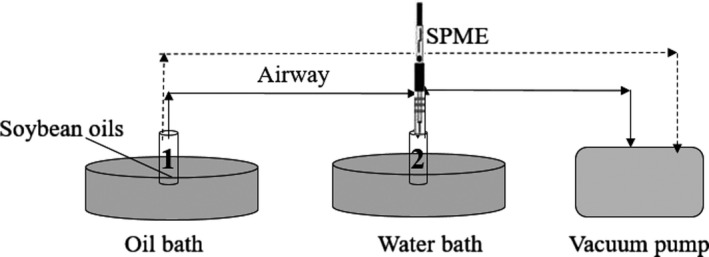
Schematic diagram of volatile matter extraction

Samples were heated in a step‐by‐step heating process. And the heating temperature point was set at 30, 60, 90, 120, 150, and 180°C, taking into consideration their smoke points of soybean oil (Katragadda et al., 2010). The experimental system is shown in Figure 1. This system consists of an oil bath and water bath (DF‐101S Heat‐gathering Style Magnetism Mixer) to control temperature and a vacuum pump to produce negative pressure and to remove the original gases. An extraction bottle numbered 1 containing 0.1 g soybean oil sample was heated in an oil bath at 30°C and balanced for 40 min to make each volatile substance to reach its saturated vapor pressure. An empty extraction bottle numbered 2 was placed in a water bath and vacuumized for 5 min by using a vacuum pump to −0.1 Mpa. The volatile compounds in the extraction bottle numbered 1 were introduced in the extraction bottle numbered 2 under negative pressure for 3 min. The volatile flavor compounds in the bottle numbered 2 were extracted using SPME fiber (50/30 µm DVB/CAR/PDMS; Supelco, Co.) for 1 hr. After extraction, the SPME with volatile compounds were immediately introduced into the GC‐MS for detection. Then, bottle numbered 1 was vacuumized (−0.1 Mpa) for 5 min to remove the residual volatile compounds with aims to eliminate interference caused by volatile flavor compounds generated during the previous temperature treatment and heated to 60°C. Then, the procedure mentioned above was repeated for the following temperature setting points.

### 2.3 GC‐MS analysis

An Agilent 6890/5975C GC‐MS system equipped with HP‐5 ms column (30 m × 0.25 mm i.d., 0.25 μm film thickness; J&W Scientific) was used to analyze volatile compounds that accumulated on the SPME fiber. The carrier gas was helium with splitless mode, which was delivered at a linear velocity of 1 ml/min. The desorption time was 5 min in the injection port at 250°C. The temperature was programmed to be hold at 35°C for 3 min and increased to 280°C at a rate of 5°C/min. The mass selective detector was operated in the electron impact ionization mode at 70 eV, in the scan range *m/z* 40–400. The interface temperature was 230°C, and the retention time of each volatile was converted to the Kovats retention index using n‐alkanes (Sigma, Co.) as references. The volatile compounds were tentatively identified by matching the mass spectra with the spectra of the reference compounds in both the Wiley mass spectra (MS) library (8th edn) and the NIST/EPA/NIH MS library (version 2.2a) and verified on the basis of mass spectra obtained from the literature and comparison of Kovats retention indices with those reported in the literature. Finally, identification accuracy was determined by separating relevant standard compounds through GC‐MS analysis under the same conditions (Dong et al., 2013).

### 2.4 Statistical analysis

The experiments for determination of volatile flavor compounds of soybean oil in the heating process were repeated for three times. Data were presented as mean ± standard error. The statistical analysis was performed using SPSS 16.0 software (SPSS Inc.). Differences between means were evaluated by one‐way analysis of variance (Duncan's multiple‐range test). Comparisons that yielded *p* < .05 were considered significant. PCA (principal component analysis) was performed using Unscrambler 9.7 (CAMO Software AS) in order to group the samples according to the class of volatiles.

## 3 RESULTS AND DISCUSSIONS

### 3.1 Compositions of volatile compounds of soybean oil in the heating process

**Figure 2:**
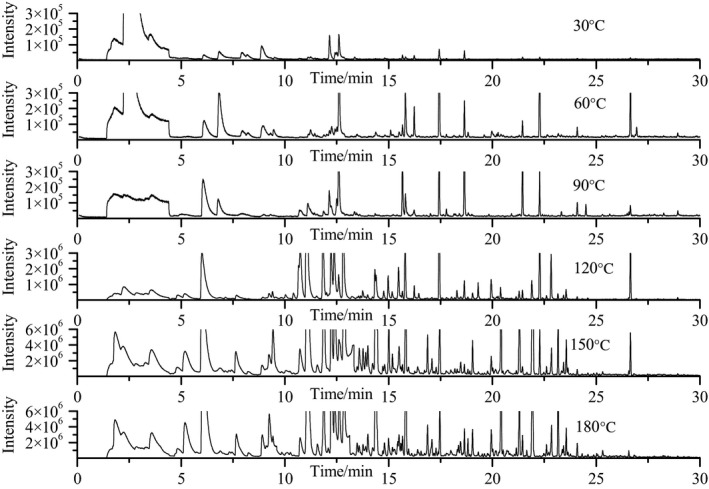
Total ion current chromatogram of soybean oil heated at different temperatures based on GC‐MS

As shown in Table 1, a total of the 72 volatile compounds including 27 aldehydes, 14 alcohols, 10 ketones, 6 furans, 9 aromatic compounds, 6 acids, and esters were detected and identified during the whole heating process. Total ion current chromatograms based on GC‐MS are shown Figure 2. The content of most volatile compounds changed significantly when the temperature increased. Six temperatures points (30, 60, 90, 120, 150, and 180°C) were selected during the heating process, and a total of 11, 19, 17, 40, 43, and 46 volatile compounds of soybean oil were identified at these temperature points, respectively.

**Table 1:** Volatile compounds from soybean oil during heating process

No.	Compound	RI	Method of identification	GC area at different heating temperature (×10^6^)					
				30°C	60°C	90°C	120°C	150°C	180°C
*Aldehydes*									
1	Butanal	593	RI, MS, STD	nd	nd	nd	255.04 ± 42.42	nd	nd
2	(E)‐2‐Butenal	647	RI, MS, STD	nd	nd	nd	85.27 ± 18.26^c^	292.40 ± 29.17^a^	149.80 ± 16.17^b^
3	Pentanal	699	RI, MS, STD	nd	nd	nd	224.01 ± 32.37^c^	1,133.31 ± 117.48^a^	944.44 ± 59.90^b^
4	(E)‐2‐Pentenal	754	RI, MS, STD	nd	nd	3.67 ± 2.29^c^	54.20 ± 9.99^b^	229.04 ± 27.79^a^	213.05 ± 17.70^a^
5	Hexanal	800	RI, MS, STD	5.62 ± 0.28^f^	15.49 ± 8.82^e^	34.91 ± 5.61^d^	493.56 ± 101.29^c^	2,352.96 ± 370.06^b^	2,855.81 ± 209.76^a^
6	(E)‐2‐Hexenal	854	RI, MS, STD	nd	nd	nd	48.31 ± 19.70^c^	467.99 ± 37.12^a^	397.69 ± 5.41^b^
7	(Z)‐4‐Heptenal	900	RI, MS, STD	nd	nd	nd	9.11 ± 1.57^b^	nd	85.90 ± 7.41^a^
8	Heptanal	901	RI, MS, STD	nd	1.99 ± 0.47^d^	1.92 ± 0.73^d^	64.02 ± 10.10^c^	312.77 ± 41.29^b^	555.61 ± 28.60^a^
9	(E,E)‐2,4‐Hexadienal	911	RI, MS, STD	nd	nd	nd	19.92 ± 4.25	nd	nd
10	(Z)‐2‐Heptenal	958	RI, MS, STD	0.88 ± 0.09^d^	1.24 ± 0.47^d^	8.29 ± 0.94^c^	765.19 ± 131.18^b^	3,326.10 ± 135.39^a^	3,265.55 ± 162.42^a^
11	Octanal	1,003	RI, MS, STD	nd	nd	nd	nd	413.69 ± 42.68^b^	642.63 ± 60.46^a^
12	(E,E)‐2,4‐Heptadienal	1,012	RI, MS, STD	nd	nd	1.61 ± 0.54^d^	322.57 ± 40.56^c^	1758.02 ± 139.16^a^	1,235.37 ± 254.22^b^
13	(E)‐2‐Octenal	1,060	RI, MS, STD	nd	nd	0.51 ± 0.09^d^	67.01 ± 6.70^c^	1,255.50 ± 84.98^b^	1,404.29 ± 184.02^a^
14	Nonanal	1,104	RI, MS, STD	0.28 ± 0.08^f^	8.63 ± 2.99^d^	4.71 ± 1.66^e^	161.03 ± 24.41^c^	598.34 ± 122.72^b^	1,288.42 ± 210.22^a^
15	(E,E)‐2,4‐Octadienal	1,115	RI, MS, STD	nd	nd	nd	nd	80.77 ± 10.16	nd
16	(Z)‐2‐Nonenal	1,148	RI, MS, STD	nd	nd	nd	10.99 ± 0.93^c^	87.27 ± 10.87^b^	103.88 ± 27.98^a^
17	(E)‐2‐Nonenal	1,162	RI, MS, STD	nd	nd	nd	nd	nd	232.19 ± 56.22
18	Decanal	1,206	RI, MS, STD	0.25 ± 0.02^e^	2.39 ± 0.90^d^	1.31 ± 0.14^d^	7.94 ± 1.14^c^	27.66 ± 3.91^b^	70.48 ± 10.39^a^
19	2,4‐Nonadienal	1,213	RI, MS, STD	nd	nd	nd	20.75 ± 2.99^c^	141.10 ± 13.69^a^	116.81 ± 40.53^b^
20	(E,E)‐2,4‐Nonadienal	1,216	RI, MS, STD	nd	nd	nd	42.07 ± 20.51	nd	nd
21	(Z)‐2‐Decenal	1,252	RI, MS, STD	nd	nd	nd	nd	28.72 ± 3.37	nd
22	(E)‐2‐Decenal	1,263	RI, MS, STD	nd	1.17 ± 0.28^d^	nd	26.29 ± 4.11^c^	403.35 ± 31.08^b^	502.47 ± 71.37^a^
23	Undecanal	1,307	RI, MS, STD	nd	nd	nd	6.45 ± 1.99^c^	19.49 ± 3.11^b^	36.91 ± 9.52^a^
24	(E,E)‐2,4‐Decadienal	1,317	RI, MS, STD	nd	nd	nd	40.50 ± 3.16^c^	1,090.23 ± 69.41^a^	635.00 ± 83.73^b^
25	2‐Undecenal	1,367	RI, MS, STD	nd	nd	nd	15.51 ± 3.86^c^	191.77 ± 3.6^b^	1,123.89 ± 279.95^a^
26	Dodecanal	1,409	RI, MS, STD	nd	0.87 ± 0.15^b^	nd	nd	13.16 ± 3.71^a^	18.24 ± 8.06^a^
27	Tridecanal	1,512	RI, MS, STD	nd	nd	nd	nd	nd	14.96 ± 6.03
*Alcohols*									
28	1‐Penten‐3‐ol	684	RI, MS, STD	nd	nd	nd	nd	269.77 ± 35.26^a^	177.99 ± 11.19^b^
29	1‐Pentanol	765	RI, MS, STD	nd	nd	nd	96.71 ± 18.38^b^	784.92 ± 129.16^a^	804.00 ± 56.39^a^
30	3‐Methyl‐1‐pentanol	838	RI, MS	nd	nd	nd	19.76 ± 5.99^b^	57.25 ± 7.91^a^	nd
31	2‐Hexyn‐1‐ol	847	RI, MS	nd	nd	nd	11.15 ± 1.77^c^	79.73 ± 8.37^a^	64.49 ± 6.40^b^
32	1‐Hexanol	868	RI, MS, STD	nd	nd	nd	10.51 ± 1.49^c^	110.40 ± 14.47^a^	83.82 ± 8.37^b^
33	4‐Methyl‐cyclohexanol	928	RI, MS	nd	nd	nd	40.71 ± 6.77	nd	nd
34	1‐Heptanol	970	RI, MS, STD	nd	nd	nd	nd	30.42 ± 10.14^b^	138.33 ± 18.13^a^
35	(E)‐2‐Hepten‐1‐ol	978	RI, MS	6.42 ± 1.02^d^	1.37 ± 0.50^e^	2.59 ± 0.53^e^	268.00 ± 54.98^c^	995.19 ± 195.52^a^	554.09 ± 55.53^b^
36	2‐Ethyl‐1‐hexanol	1,030	RI, MS	nd	1.93 ± 0.44^a^	1.16 ± 0.37^a^	nd	nd	nd
37	2,4‐Dimethyl‐cyclohexanol	1,032	RI, MS	nd	nd	nd	10.04 ± 1.14^c^	187.96 ± 24.66^a^	84.72 ± 13.03^b^
38	3,5‐Octadien‐2‐ol	1,038	RI, MS	nd	nd	nd	67.01 ± 6.70	nd	nd
39	1‐Octanol	1,071	RI, MS, STD	nd	nd	nd	nd	69.88 ± 8.21^b^	90.96 ± 15.41^a^
40	2,6‐Dimethyl‐1,7‐octadien‐3‐ol	1,095	RI, MS	nd	nd	nd	101.23 ± 9.59^b^	182.80 ± 25.17^a^	106.18 ± 47.51^b^
41	(6Z)‐Nonen‐1‐ol	1,171	RI, MS	nd	nd	nd	10.26 ± 1.01	nd	nd
*Ketones*									
42	2‐Butanone	587	RI, MS, STD	nd	nd	nd	nd	1,179.07 ± 122.92^a^	793.59 ± 160.66^b^
43	2‐Hexanone	790	RI, MS, STD	nd	nd	nd	nd	nd	74.12 ± 2.86
44	3‐Hexen‐2‐one	845	RI, MS	nd	nd	nd	nd	53.80 ± 5.88	nd
45	2‐Heptanone	891	RI, MS, STD	nd	nd	5.14 ± 2.72^d^	20.35 ± 6.50^c^	161.80 ± 14.01^b^	276.84 ± 16.67^a^
46	2,3‐Octanedione	984	RI, MS	nd	nd	nd	24.61 ± 6.85	nd	nd
47	3‐Octanone	986	RI, MS, STD	nd	nd	nd	nd	nd	81.10 ± 2.32
48	3‐Octen‐2‐one	1,040	RI, MS	nd	nd	nd	nd	145.39 ± 15.73^a^	68.30 ± 8.01^b^
49	(E,E)‐3,5‐Octadien‐2‐one	1,073	RI, MS	0.68 ± 0.06^b^	nd	nd	32.04 ± 4.16^a^	nd	nd
50	3‐Nonen‐2‐one	1,142	RI, MS	nd	nd	nd	8.26 ± 0.22^b^	157.83 ± 16.29^a^	148.66 ± 31.52^a^
51	(E)‐ 6,10‐Dimethyl‐5,9‐undecadien‐2‐one,	1,453	RI, MS	nd	0.71 ± 0.28^b^	nd	1.76 ± 0.35^a^	nd	nd
*Furans*									
52	Tetrahydro‐2‐methyl‐furan	674	RI, MS	nd	nd	nd	nd	124.07 ± 8.85	nd
53	2‐Pentylfuran	993	RI, MS, STD	nd	4.38 ± 0.54^d^	3.28 ± 0.59^d^	178.29 ± 40.51^c^	616.02 ± 52.02^b^	1534.55 ± 184.75^a^
54	5‐Ethyldihydro‐2(3H)‐furanone	1,057	RI, MS	nd	nd	nd	nd	81.12 ± 7.20	nd
55	Dihydro‐3‐methylene‐5‐methyl‐2‐furanone	1,075	RI, MS	nd	nd	nd	65.48 ± 7.14^c^	260.86 ± 35.61^a^	120.35 ± 11.38^b^
56	5‐Butyldihydro‐2(3H)‐furanone	1,261	RI, MS	nd	nd	nd	nd	nd	19.80 ± 8.71
57	2‐n‐Octylfuran	1,297	RI, MS	nd	nd	nd	nd	nd	278.84 ± 113.07
*Aromatic compounds*									
58	Toluene	763	RI, MS, STD	1.27 ± 0.15^a^	1.69 ± 0.60^a^	nd	nd	nd	nd
59	Ethylbenzene	855	RI, MS, STD	5.38 ± 0.63^a^	6.92 ± 1.86^a^	nd	nd	nd	nd
60	p‐Xylene	865	RI, MS, STD	4.13 ± 0.16^a^	5.03 ± 0.68^a^	2.15 ± 0.60^b^	nd	nd	nd
61	Styrene	893	RI, MS, STD	11.46 ± 0.93^a^	14.09 ± 3.55^a^	nd	nd	nd	nd
62	Pentyl‐benzene	1,157	RI, MS	nd	nd	nd	nd	nd	22.77 ± 2.18
63	Naphthalene	1,182	RI, MS	nd	1.00 ± 0.38^a^	0.73 ± 0.48^b^	nd	nd	nd
64	2‐Methyl‐naphthalene	1,298	RI, MS	nd	1.05 ± 0.28^a^	0.90 ± 0.38^a^	nd	nd	nd
65	1‐Methyl‐naphthalene	1,307	RI, MS	nd	nd	0.77 ± 0.43	nd	nd	nd
66	2,4‐di‐tert‐butylphenol	1,519	RI, MS	nd	0.98 ± 0.41^b^	3.03 ± 0.08^a^	4.31 ± 2.59^a^	nd	nd
*Acids and esters*									
67	Butyrolactone	915	RI, MS	nd	nd	nd	nd	nd	123.21 ± 28.60
68	Butanoic acid, butyl ester	995	RI, MS, STD	1.62 ± 0.22^a^	1.94 ± 0.65^a^	nd	nd	nd	nd
69	Hexanethioic acid, S‐methyl ester	1,063	RI, MS	nd	nd	nd	nd	159.51 ± 19.52	nd
70	Heptanoic acid	1,078	RI, MS	nd	nd	nd	nd	nd	58.97 ± 22.80
71	Nonanoic acid	1,273	RI, MS	nd	nd	nd	nd	89.39 ± 58.74^a^	64.86 ± 18.09^b^
72	Hexanoic acid, pentyl ester	1,287	RI, MS	nd	nd	nd	6.88 ± 2.23^c^	22.43 ± 3.23b	37.24 ± 12.70^a^

Abbreviations: MS, identification by MS spectra; nd, Compound not detected in the sample; RI, Kovat's retention indexes; STD, comparison with a standard compound.

Letters like a,b,c,d,e,f at the top right corner of values mean statistical differences.

Aldehydes are the most varied whether in type or in relative content among these volatile compounds. Most aldehydes are formed above 90°C. During the whole heating process, we identified 10 alkanals from C4 to C13 (butanal, pentanal, hexanal, heptanal, octanal, nonanal, decanal, undecanal, dodecanal, and tridecanal); 10 alkenals from C4 to C11 including isomers ((E)‐2‐butenal, (E)‐2‐pentenal, (E)‐2‐hexenal, (Z)‐4‐heptenal, (Z)‐2‐heptenal, (E)‐2‐octenal, (Z)‐2‐nonenal, (Z)‐2‐decenal, (E)‐2‐decenal, and 2‐undecenal), and six alkadienals from C6 to C10 including isomers ((E, E)‐2,4‐hexadienal, (E, E)‐2, 4‐heptadienal, (E, E)‐2, 4‐octadienal, 2, 4‐nonadienal, (E, E)‐2, 4‐nonadienal, and (E, E)‐2, 4‐decadienal). Meanwhile, most of aldehydes from C6 to C10 are the main product in the early stage of the heating process, and C10–C12 aldehydes were gradually detected as the temperature raised.

It was commonly accepted that aldehydes were original from the lipid oxidation (Varlet, Prost, & Serot, 2007). If the reaction begins, it will always be there until the oxygen runs out. Therefore, some compounds exist throughout the heating process, for example, hexanal, nonanal, decanal, and (Z)‐2‐heptenal (Table 1). Heptanal, (E)‐2‐decenal, and dodecanal appeared at 60°C, while (E)‐2‐pentenal, (E, E)‐2, 4‐heptadienal, and (E)‐2‐octenal appeared with an increase in temperature up to 90°C. The relative content of certain compounds including pentanal, hexanal, nonanal, (Z)‐2‐heptenal, (E)‐2‐octenal, (E, E)‐2, 4‐heptadienal, and (E, E)‐2, 4‐decadienal were relatively high at high temperatures (Table 1), suggesting they may play an important role in soybean oil aromas. According to previous reports, hexanal has a green grass flavor, nonanal has green grass flavor and a fatty scent (Ahmed, Dennison, Dougherty, & Shaw, 1978; Guadagni, Buttery, & Okano, 1963), and 2‐octanal has a coffee and nut flavor (Furia, Bellanca, Furia, & Bellanca, 2010). Some researchers previously found that these compounds are mainly derived from the oxidation of oleic acid, linoleic acid, and linolenic acid. Among the aldehyde substances, nonanal mainly comes from oleic acid, while pentanal, hexanal, 2‐octanal, and 2‐ and 4‐decadienal mainly come from linoleic acid; 2, 4‐heptanal comes from linolenic acid, while 2‐heptenal has not been detected (Frankel, 1983; Fujisaki, Endo, & Fujimoto, 2002; Shen, Fehr, Johnson, & White, 1996). This result was consistent with the rich linoleic acid content of soybean oil, followed by oleic acid and linolenic acid.

Alcohols are another important class of compounds that make up the volatile flavor of soybean oil during heating process. As it is showed in Table 1, most of alcohols are detected above 120°C. Only, (E)‐2‐hepten‐1‐ol was detected throughout the whole heating process. Alcohols are also considered as the product of fatty acid oxidation (Dong et al., 2013). 1‐penten‐3‐ol has an irritating buttery taste, 1‐pentanol has a floral and resinous taste, and 1‐hexanol has the smell of grass and flowers.

Both ketone and furan compounds are formed at high temperatures, typically above 120°C, except for 2‐heptanone, (E, E)‐3, 5‐octadien‐2‐one, and 2‐pentylfuran, etc. The relative amount of 2‐butanone and 2‐pentylfuran is abundant at high temperatures of 150 or 180°C. Furan compounds were usually considered to be products of Maillard reaction (Dong et al., 2015).

Most aromatic compounds were detected at <60°C. The reason for this is still unknown. Some acids and esters containing <10 carbon atoms were detected at high temperatures, such as heptanoic and nonanoic acid. Although the amount of esters detected in the process is small, they often bring pleasant, sweet, fruity odors.

Generally, it is considered that most of volatile compounds were original from the lipid oxidation, and the major precursor is lipid hydroperoxides, including alkyl hydroperoxides, allyl hydroperoxides, and fatty ester hydroperoxides. In the lipid oxidation process, these precursors were firstly formed, followed by forming corresponding free radical, which made the lipid schemes decomposed by free radical oxidation, yielding volatile aldehydes. Taking oleate oxidation for example, a mixture of four positional 8‐,9‐,10‐, and 11‐hydroperoxides were detected during this process, which resulted in forming 2‐undecenal, 2‐decenal, octanal, nonanal, and decanal.

### 3.2 Characteristics of volatile compounds composition of soybean oil at different heating temperature

**Figure 3:**
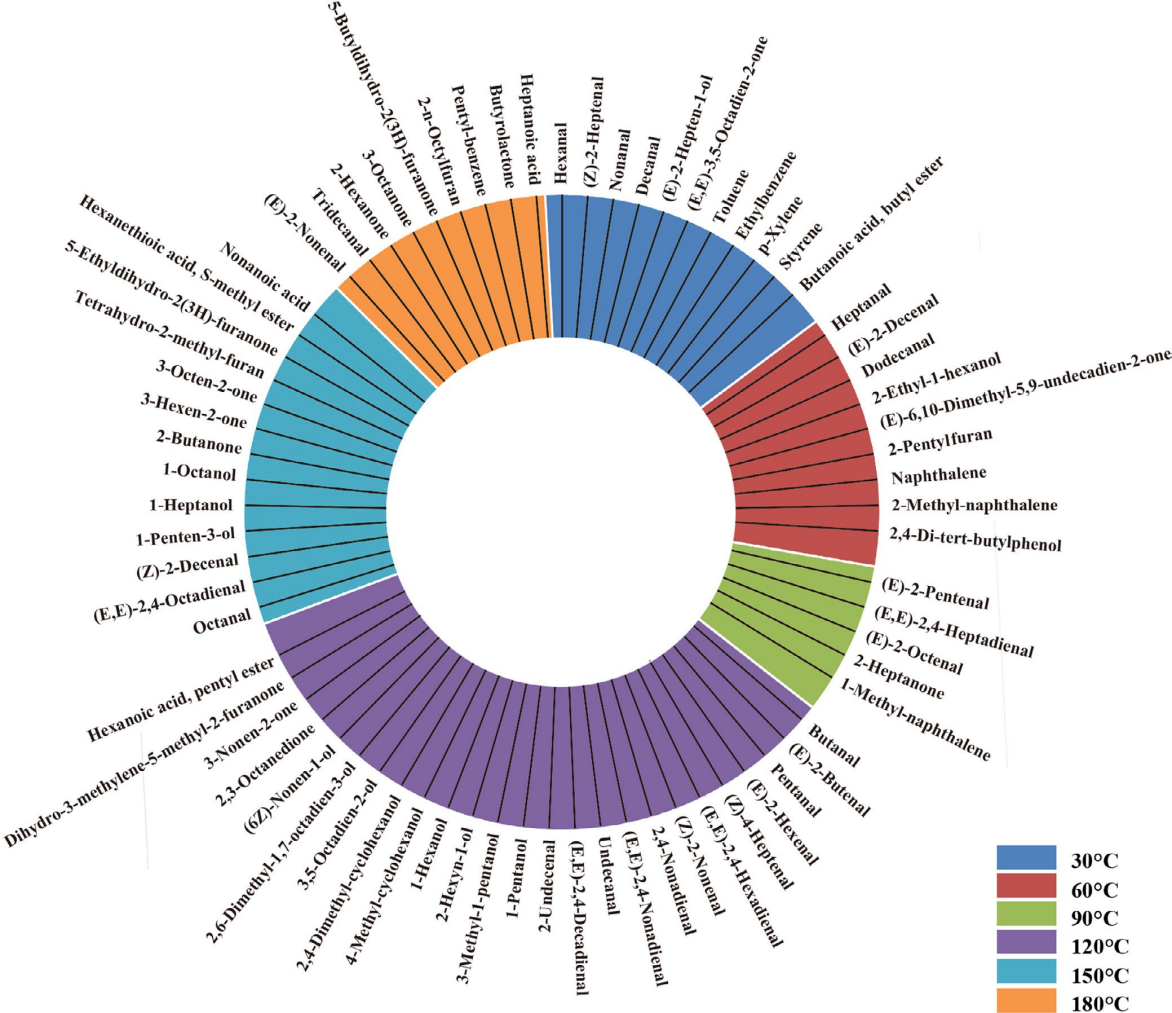
The forming temperature point of different volatile compounds

To visualize the formation temperature of different volatiles, the volatile composition of soybean oil at different heating temperature point was shown in Figure 3. The general trends indicated that aromatic compounds and a small amount of aldehydes formed at lower temperatures; while a large number of aldehydes, alcohols, ketones, and acid ester compounds formed at higher temperatures. A total of 11 volatiles were identified at 30°C during heating, including aldehydes (4), ketones (1), alcohols (1), aromatics (4), acid, and ester (1). However, it does not mean that these compounds were generated at this temperature point, but the basic volatile ingredients of the soybean oil.

With the temperature raised, there were nine and five volatiles generated at 60 and 90°C, respectively. As shown in Figure 3, they were heptanal, (E)‐2‐decenal, dodecanal, 2‐ethyl‐1‐hexanol, 6,10‐dimethyl‐5,9‐undecadien‐2‐one, 2‐pentylfuran, naphthalene, 2‐methy‐naphthalene, and 2,4‐di‐tert‐butylphenol generated at 60°C, (E)‐2‐pentenal, (E,E)‐2,4‐heptadienal, (E)‐2‐octenal, 2‐heptanone, and 1‐methy‐naphthalene generated at 90°C. Most of these volatiles are aldehyde with a carbon chain below 10.

**Figure 4:**
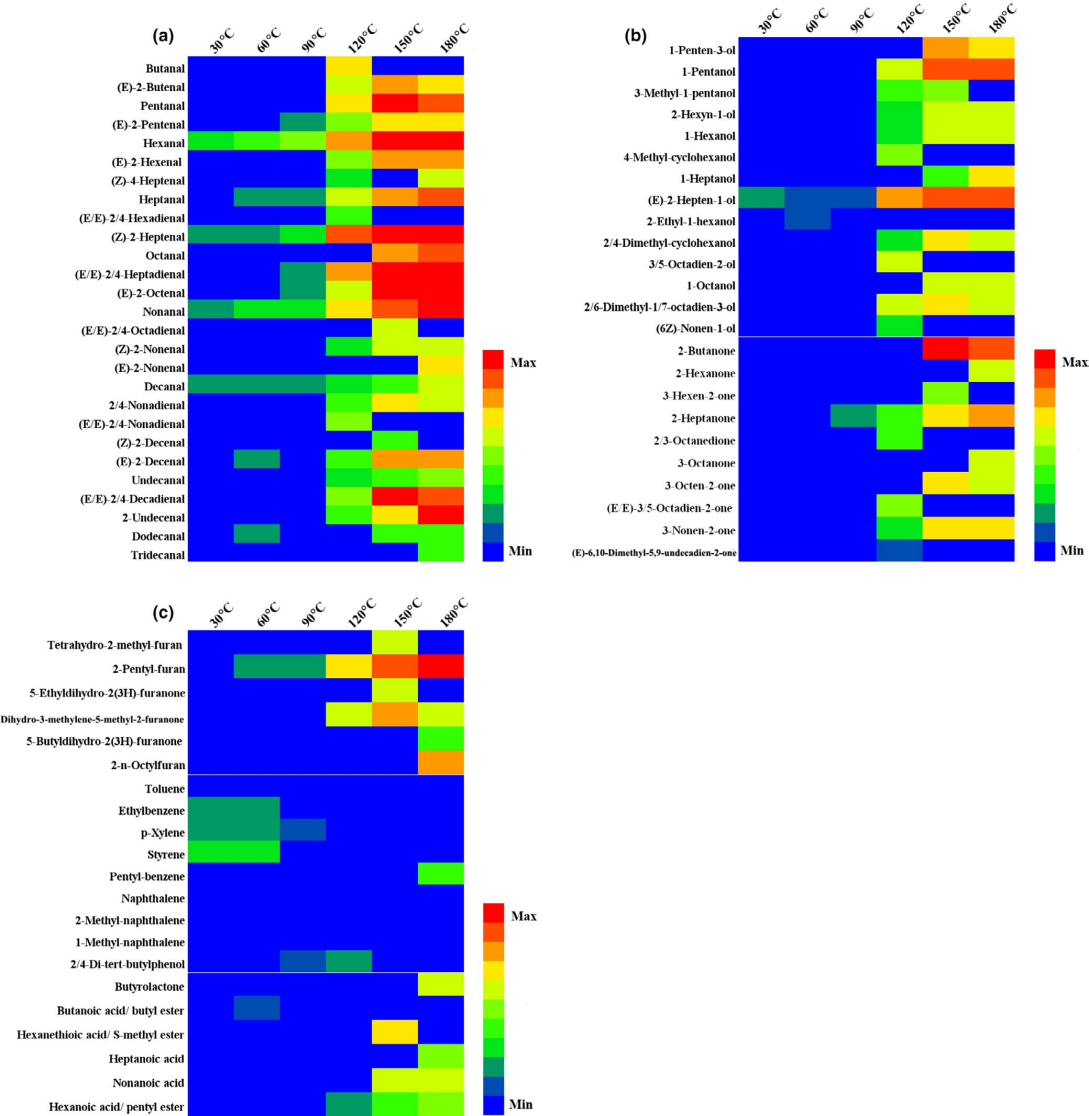
Heat map of volatile compounds of soybean oil in the heating process. (a) Aldehydes, (b) alcohols and ketones (c) furans, aromatic compounds, acids, and esters

In order to see the variation tendency of compounds more intuitively at different temperatures, a heat map was drawn for the volatile flavor compounds of soybean oil. The results are shown in Figure 4. The color of the heat map gradually changes from cold to warm. The cold color represents a lower content, and the warm color represents a relative higher content. Figure 4a–c shows heat map of aldehydes, alcohols and ketones, furans, aromatic compounds, and acids esters, respectively. It also can be seen from Figure 4, the color of most volatile compounds changed gradually from cold to warm with temperature increased, except aromatic compounds.

As shown in Figure 3, there were 25 volatile compounds generated at 120°C, including aldehydes (12), alcohols (9), and ketones (3), which has the largest generated number of aldehydes and alcohols compounds during heating, such as pentanal, (E)‐2‐hexenal, and (E,E)‐2‐4‐decadienal. According to previous researches, odor characteristics of the frying soybean oil were mainly correlated with some aldehydes (Katragadda et al., 2010; Wu & Chen, 1992). Buttery odor and overall odor quality of oil inversely correlated with hexanal, (E)‐2‐hexenal, heptanal, (Z)‐2‐heptenal, and 2‐pentylfuran. Rancid and painty odors of oil were correlated with pentanal. Fishy and beany odor were moderately correlated with (E,E)‐2‐4‐decadienal. Grassy, rancid, painty, and acrolein odors of oil were positively correlated with hexanal, (E)‐2‐hexenal, heptanal, (Z)‐2‐heptenal, and 2‐pentylfuran. And buttery and rancid odors were considered as the best indicators of overall odor quality (Brewer, Vega, & Perkins, 1999). Moreover, hexanal, a known lipid oxidation breakdown product, has been used as an indicator of rancidity in a variety of foods; the height of the hexanal peak in a gas chromatogram has been well‐correlated with sensory evaluation for rancid (Brewer et al. 1999). Meanwhile, the concentration of hexanal, heptanal, (Z)‐2‐heptenal, and 2‐pentylfuran also increased dramatically at this temperature point (Figure 4). Consequently, 120°C is considered as a critical temperature point for the formation of soybean oil volatile compounds in the whole heating process, and the off‐flavor began to release largely. Above this temperature, there are 13 and nine volatile compounds generated at 150 and 180°C, respectively. Most of ketones are formed at 150°C, and acids are detected at 180°C.

**Figure 5:**
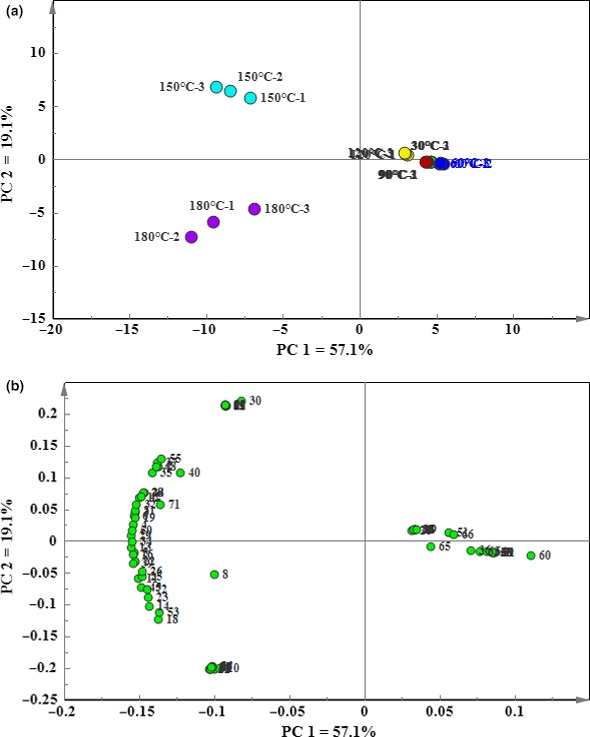
The principal component analysis (PCA) based on the relative content of volatile substances formed in the soybean oil heating process. (a) The scores plot, (b) the loading plot

In order to better understand the volatile flavor characteristics of soybean oil at different temperatures, PCA was carried out on the 72 volatile compounds of the heated samples. The peak areas of each compound were normalized, and the first and second principal components (PC1 and PC2) were chosen ultimately. The score scatter and loading scatter plots are shown in Figure 5a,b, respectively.

It can be seen from Figure 5a that the cumulative proportion for PC1 (explained variation [57.1%] and PC2 [19.1%] were 76.2%). The scores of soybean oil heated at lower temperature (30, 60, and 90°C) were close to each other and even overlapped, mainly concentrated in the positive loading region of PC1, indicating that the flavor composition below 90°C was similar. Corresponding to the loadings scatter plots (Figure 5b), 2‐ethyl‐1‐hexanol, (E)‐6,10‐dimethyl‐5, 9‐undecadien‐2‐one, p‐xylene, 1‐methyl‐naphthalene, and 2,4‐di‐tert‐butylphenol formed a cluster at lower temperatures, indicating that these compounds contribute greatly to the flavor composition of soybean oils at lower temperatures. It also shows that these compounds are detected below 90°C.

It also can be seen from Figure 5a that the scoring points at the higher temperatures (150 and 180°C) and the lower temperature (60, 90, and 120°C) can be clearly separated. They located in the positive and negative region of PC2, respectively. This means that flavor composition of the soybean oil has a big difference between higher and lower temperature. Corresponding to the loadings scatter plots (Figure 5b), the majority of volatile compounds located in this region, which further identify that most of volatile compounds are formed at higher temperatures.

Generally, volatile compounds from soybean oil come from two main lipid oxidation reactions, including photo oxidation and thermal oxidation. Photo oxidation is a slow oxidation process, in which the forming of volatile compounds are both temperature‐dependent and time‐dependent. Inversely, thermal oxidation is a fast oxidation process. There is no possibility for volatiles forming to be time‐dependent because of the rapid raised temperature. And based on the data in this paper, the formation of volatiles shows a certain temperature‐dependent trend. As a result, authors consider that volatiles forming of soybean oil are temperature‐dependent in the heating process.

## 4 CONCLUSION

Oil heating or cooking process is a very quick and complex network, during which lots of sharp oxidative reactions occurred rapidly. Temperature played an important role in the soybean oil flavor forming process, which shows a temperature‐dependent trend during heating, and more evidences were needed to draw this conclusion in the future. Meanwhile, the forming temperature of each volatile was finally determined. Subsequently, this research provides a theoretical possibility to modulate flavor production by controlling the temperature in the heating process.

## CONFLICT OF INTEREST

We declare that we have no conflicts of interest.

## ETHICAL APPROVAL

This study does not involve any human or animal testing.
